# AdipoRon Inhibits Neuroinflammation Induced by Deep Hypothermic Circulatory Arrest Involving the AMPK/NF-κB Pathway in Rats

**DOI:** 10.3390/pharmaceutics14112467

**Published:** 2022-11-15

**Authors:** Weidong Yan, Sizhe Gao, Qiaoni Zhang, Jiachen Qi, Gang Liu, Yuan Teng, Jian Wang, Shujie Yan, Bingyang Ji

**Affiliations:** 1 Department of Cardiopulmonary Bypass, Fuwai Hospital, National Center for Cardiovascular Disease, State Key Laboratory of Cardiovascular Medicine, Chinese Academy of Medical Sciences & Peking Union Medical College, Beijing 100037, China; 2 Department of Pain Medicine, Beijing Tsinghua Changgung Hospital, School of Clinical Medicine, Tsinghua University, Beijing 102218, China

**Keywords:** deep hypothermic circulatory arrest, AdipoRon, neuroinflammation

## Abstract

Deep hypothermic circulatory arrest (DHCA) can induce systemic inflammatory response syndrome, including neuroinflammation. Finding suitable compounds is necessary for attenuating neuroinflammation and avoiding cerebral complications following DHCA. In the present study, we established DHCA rat models and monitored the vital signs during the surgical process. After surgery, we found significantly increased proinflammatory cytokines (IL-6, IL-1β, and TNF-α) in DHCA rats. Quantitative proteomics analysis was performed for exploring the differentially expressed proteins in hippocampus of DHCA rats and the data showed the adiponectin receptor 1 protein was upregulated. More importantly, administration of AdipoRon, a small-molecule adiponectin receptor agonist, could improve the basic vital signs and attenuate the increased IL-6, IL-1β, and TNF-α in DHCA rats. Furthermore, AdipoRon inhibits the activation of microglia (M1 state) and promotes their transition to an anti-inflammatory state, via promoting the phosphorylation of adenosine monophosphate-activated protein kinase (AMPK), and downregulating nuclear factor kappa B (NF-κB) in DHCA rats. Consistently, we used LPS-treated BV2 cells to mimic the neuroinflammatory condition and found that AdipoRon dose-dependently decreased cytokines, along with increased phosphorylation of AMPK and downregulated NF-κB. In conclusion, our present data supported that AdipoRon inhibited DHCA-induced neuroinflammation via activating the hippocampal AMPK/NF-κB pathway.

## 1. Introduction

Cardiopulmonary bypass (CPB) is a routine practice in clinical cardiac surgery. Based on CPB, deep hypothermic circulatory arrest (DHCA) can provide better bloodless surgical fields for complex surgeries such as cardiac surgery, pulmonary endarterectomy, and surgeries for removing renal tumors [1,2]. The DHCA procedure can reduce brain metabolism and oxygen requirements, and it has traditionally provided cerebral protection for many decades. However, the risk of cerebral cellular injury associated with DHCA still needs attention. In the clinic, DHCA is an independent risk factor for post-operative headaches [3]. Recent studies revealed that DHCA can induce cerebral mitochondrial dysfunction and alter neonatal neurodevelopmental progression [4,5]. Further, the systemic inflammatory response syndrome (SIRS) occurs commonly in patients with DHCA [6]. The surgical trauma, the ischemia/reperfusion (I/R) injury, and the exposure to artificial surfaces during DHCA might induce SIRS [7,8]. The poorly controlled neuroinflammation following SIRS could trigger severe cerebral complications, including cerebral infarction, hydrocephalus, and neuronolyticdemyelination [9]. It has been reported that the incidence of cerebral complications after DHCA was 26.72% [10], and there are only limited drugs that can prevent it in clinics. Therefore, there is an urgent to find suitable compounds to attenuate neuroinflammation and improve prognosis following DHCA.

AdipoRon, a small-molecule adiponectin receptor agonist, was discovered in 2013 [11]. Adiponectin is well studied and is considered protective based on its potent anti-lipotoxic, anti-apoptotic, and anti-inflammatory actions [12]. Similar to the natural adiponectin, AdipoRon also shows anti-inflammatory effects by activating the adenosine monophosphate-activated protein kinase (AMPK) signaling pathway [13]. Several studies have conferred to AdipoRon marked anti-cancer properties in pancreatic ductal adenocarcinoma, and ovarian cancer [14,15,16]. Furthermore, AdipoRon acts as an anti-proliferative molecule in osteosarcoma cell lines [17]. In the cardiovascular system, AdipoRon can delay cardiac remodeling, attenuate post-ischemic cardiac injury, and improve cardiocirculatory recovery after DHCA. More importantly, AdipoRon can penetrate the blood–brain barrier and shows neuroprotective effects [18]. It can prevent glutamate-induced cell death, attenuate neuroinflammation after intracerebral hemorrhage, and suppress macrophage recruitment after spinal cord injury [19,20,21]. However, whether AdipoRon treatment could alleviate DHCA-induced neuroinflammation remains obscure.

The nuclear factor kappa B (NF-κB) is a transcriptional factor which can promote the proinflammatory M1 polarization of microglia [22]. Our previous study has demonstrated that the activation of NF-κB was associated with neuroinflammation following DHCA [23]. Interestingly, the natural adiponectin could also inhibit NF-κB and thus suppress the activation of C reaction protein, which indicates an acute inflammation [24]. Therefore, we hypothesized that AdipoRon could alleviate neuroinflammation following DHCA, which may be related to the activation of AMPK and/or inhibition of NF-κB. This is the first study to investigate the effects of AdipoRon on DHCA-related neuroinflammation in vivo and in vitro. Our data will provide a basis for the clinical usage of AdipoRon for improving the outcome of patients undergoing DHCA in cardiac surgery in the future.

## 2. Materials and Methods

### 2.1. Animals

Male Sprague Dawley (SD) rats (weighted 500–600 g, age 12–14 weeks, obtained from the HFK Bioscience, China) were kept under standard laboratory conditions, with free access to food and water (by Fuwai Animal Center). Rats were randomly allocated into 3 groups: the sham group (n = 11), the DHCA group (n = 11) and the AdipoRon-treated DHCA group (n = 6). A total of 5 rats in both the sham group and DHCA group were randomly selected for the proteomic analysis of the hippocampus.

### 2.2. Cardiac Surgery with CPB/DHCA

Briefly, fasting rats were randomly assigned to different groups. In the DHCA and DHCA + AdipoRon groups, rats were anesthetized with sevoflurane (2–3%), incubated and the left femoral artery was cannulated to monitor the mean arterial blood pressure (MAP). The tail artery and the right external jugular vein were cannulated and connected to a CPB circuit, which was primed with 12 mL of 6% hydroxyethyl starch and 2 mL saline with 250 IU heparin. Blood flow was directed from the jugular vein through silicon tubes to CPB circuits and back to the corporal circulation via the tail artery. After heparinization (500 IU/kg), CPB was initiated at a flow rate of 160–180 mL/kg/min for 10 min. The flow rate was decreased during the cooling process to a target temperature of 20 °C. After 30 min of systemic cooling, DHCA was induced for 45 min. After that, CPB was restarted and the rats were subjected to 60 min of rewarming and 60 min for the reperfusion. Then, the CPB was weaned off within 20 min. Finally, rats were ventilated for 30 min without CPB support. During the rewarming phase, norepinephrine was administrated in the case of MAP ≤ 50 mmHg. Rats in the sham group were only anesthetized, cannulated, and heparinized. At the end of the experiment, blood samples were collected and centrifuged (3000 rpm for 20 min at 4 °C) within 30 min. The upper layers of plasma were stored at −80 °C for further analysis. Euthanasia was performed by cervical dislocation under deep anesthesia. The left hippocampus was harvested in liquid nitrogen immediately and the right hippocampus was harvested into the 4% formaldehyde for further analysis.

A timeline of the experimental procedures including drug administration and sample taking is shown in Supplementary Figure S1. Baseline physiological parameters, MAP, heart rate (HR), and rectal temperature were continuously monitored throughout the procedure.

### 2.3. Proteomic Analysis of Hippocampus Based on Tandem Mass Tags (TMT)

The hippocampus samples were ground individually in liquid nitrogen and lysed with PASP lysis buffer, followed by 5 min of ultrasonication on ice. The lysate was centrifuged at 12,000× *g* for 15 min at 4 °C and the supernatant was reduced with 10 mM DTT for 1 h at 56 °C, and subsequently alkylated mixed with 4 fold the volume of precooled acetone and incubated at −20 °C for at least 2 h. Samples were then centrifuged at 12,000× *g* for 15 min at 4 °C and the precipitation was collected. After washing with 1 mL cold acetone, the pellet was dissolved by dissolution buffer. TMT analysis was conducted by Novogene Biotech Co., Ltd. (Beijing, China). Proteins with FC ≥ 1.2 or FC ≤ 0.83 and *p* value < 0.05 were considered to be differentially expressed proteins (DEPs). The target proteins were analyzed by the Kyoto Encyclopedia of Genes and Genomes (KEGG) annotation.

### 2.4. Drug Treatment

AdipoRon (12.5 mg/kg body weight, Adooq Bioscience, Irvine, CA, USA) was dissolved in 1% dimethyl sulfoxide (DMSO, Sigma-Aldrich, St. Louis, MO, USA) and administrated twice via the reservoir at time points 5 min after the initiation of CPB and with the beginning of the rewarming process, respectively. The sham group was given the same volume of DMSO.

### 2.5. Blood Gas Analysis

Artery blood samples for blood gas analysis (i-STAT, Chicago, IL, USA) were collected at 4 time points: 10 min after the initiation of CPB (T_0_), 45 min after the DHCA (T_1_), 60 min after rewarming (T_2_) and 60 min after the reperfusion (T_3_).

### 2.6. BV2 Cell Culture

Murine microglial BV2 cells were cultured in high-glucose (4.5 g/L) Dulbecco’s modified Eagle’s medium (Sigma-Aldrich, Missouri, USA), containing 10% fetal bovine serum, 1% penicillin and streptomycin in an incubator at 37 °C with 5% CO_2_ (balanced with air). After incubated in 6-well plate (1–2 × 10^5^/well) for 12 h, cells were treated with AdipoRon (0, 1, 10, 50 μM) and the vehicle control cells were treated with 1% DMSO. Cells and the culture supernatant were collected for further analysis. In order to establish an in vitro neuroinflammation model, inflammation was induced with 1 μg/mL lipopolysaccharide (LPS, Sigma-Aldrich, Missouri, USA), which was collected from Escherichia coli 0111:B4.

### 2.7. Immunostaining of Hippocampus

The 4% paraformaldehyde was used to fix the collected rat hippocampus tissue, and then they were embedded in paraffin and were sectioned at 4 μm. Immunofluorescence was used for the detection of activated microglial cells. Sections were permeabilized with 0.1% Triton X-100 (ZSGB-Bio, Beijing, China) and incubated with 10% goat serum (ZSGB-Bio, Beijing, China). Sections were further incubated with adiponectin receptor 1 (1:50, Santa Cruz, CA, USA), anti-ionized calcium-binding adaptor molecule-1 (Iba-1) antibody (1:500, Servicebio, Wuhan, China), and DC206 (1:200, Servicebio, Wuhan, China) overnight at 4 °C followed by additional incubation with Cy3-conjugated goat anti-rabbit IgG (1:300, Servicebio,Wuhan, China) for 1 h at room temperature. Nuclei were counterstained with 4′,6-diamidino-2-phenyl indole (DAPI, ZSGB-Bio, Beijing, China). Immunohistochemistry (IHC) was used for the detection of *p*-AMPK/NF-κB protein. Sections were incubated with rabbit anti-*p*-AMPK antibody (1:100, Wanleibio, Shenyang, China) and rabbit anti-NF-κB p65 antibody (1:400, Cell Signoling Technology, Danvers, MA, USA) overnight at 4 °C. Then, they were incubated with the enzyme-conjugated goat anti-rabbit IgG polymer in a Rabbit Two-step kit (ZSGB-Bio, Beijing, China). Sections were screened using Pannoramic SCAN (3D HISTECH, Budapest, Hungary) and analyzed using ImageJ software (National Institutes of Health, Bethesda, MD, USA).

### 2.8. Western Blots

Total proteins were extracted from hippocampus or cells in cold lysis buffer (cOmplete Lysis-M, EDTA free, Roche, Basel, Switzerland) supplemented with proteinase and phosphatase inhibitors (cOmplete Mini and PhosSTOP, Roche, Basel, Switzerland). The concentration of extracted proteins was determined using a BCA kit (Beyotime, Shanghai, China). Proteins were separated by 4–12% sodium dodecyl sulfate-polyacrylamide gel electrophoresis (SDS-PAGE, Invitrogen, Waltham, MA, USA) and transferred onto nitrocellulose membranes (Invitrogen, USA). Proteins were immunoblotted with primary antibodies against adiponectin receptor 1 (Catalog. #sc-518030, 1:100, Santa Cruz, CA, USA), phosphorylated AMPK-α (Catalog. #2535, 1:1000, Cell Signaling Technology, Danvers, MA, USA), AMPK-α (Catalog. #5831, 1:1000, Cell Signaling Tecnology, USA), NF-κB p65 (Catalog. #8242, 1:1000, Cell Signaling Tecnology, USA) and β-Actin (Catalog. #49900, 1:30,000, Abcam, Cambridge, UK) in combination with horseradish peroxidase-conjugated secondary-antibodies (Catalog. #111-035-003, 1:5000, Jackson ImmunoResearch, West Grove, PA, USA). Protein bands were visualized using Amersham Imager 800 (Cytiva, Marlborough, MA, USA) and quantified by ImageJ software (National Institutes of Health, USA). β-Actin/GAPDH was used as an internal control for protein inputs.

### 2.9. Enzyme-Linked Immunosorbent Assay

Enzyme-Linked Immunosorbent Assay (ELISA) was used to measure the concentration of proinflammatory cytokines. Tumor necrosis factor-α (TNF-α) and interleukin (IL)-1β and IL-6 in rat blood plasma were measured using ELISA kits (Boster, Wuhan, China). The levels of TNF-α, IL-1β, and IL-6 in the cultured medium of BV2 cells were measured using ELSIA kits (ABclonal Technology, Wuhan, China).

### 2.10. Quantitative Real-Time Polymerase Chain Reaction

Total RNAs of BV2 cell were extracted using RNAiso Plus reagent (Takara, Kyoto, Japan). RNA concentration and purity were determined with Nanodrop 2000 (Thermo, Waltham, MA, USA). Reverse transcription to cDNA was performed using RT SuperMix (Vazyme, Nanjing, China) according to the manufacturer’s instructions. Quantitative real-time polymerase chain reaction (qPCR) was performed using SYBR qPCR Master Mix (Vazyme, Nanjing, China) according to the manufacturer’s instructions. Relative expression levels were normalized to Actin as internal standard controls and they were calculated by the 2^−ΔΔCt^ method. Specific primers were designed as shown in Supplementary Table S1.

### 2.11. Nitric Oxide Production Measurement

NO^2-^ was measured in supernatants to assess the nitric oxide (NO) production in BV2 cells using a Nitric Oxide Assay Kit (Beyotime, Shanghai, China) according to the manufacturer’s instructions. The absorbance at 540 nm was measured by a microplate reader.

### 2.12. Statistical Analysis

Data were collected and statistically analyzed using Graph Pad Prism 9.0 (GraphPad, San Diego, CA, USA). Numerical data are presented as the mean ± standard error of mean (SEM). Student’s *t* test was used for 2-group analysis, and Welch’s correction was used if the data did not pass the F-test. The Mann–Whitney test was used if the data did not pass the normality test. Unpaired data of multiple groups were analyzed using one-way ANOVA followed by Bonferroni’s multiple comparison tests. A *p*-value less than 0.05 (2-tailed) was considered statistically significant.

## 3. Results

### 3.1. Adiponectin Receptor 1 Protein Was Upregulated after DHCA

**Figure 1:**
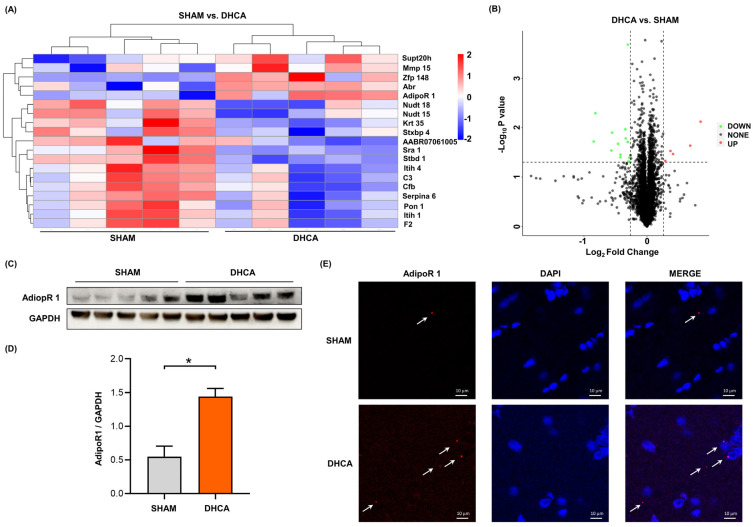
Differentially expressed proteins of the hippocampus after DHCA and the expression of adiponectin receptor 1. (**A**) Cluster analysis of differentially expressed proteins (DEPs) in DHCA rats compared with rats in the sham groups. Each group contained five biological replicates. (**B**) The volcano plot displayed DEPs. The orange and green spots indicate upregulated and downregulated proteins, respectively. Additionally, the gray spots represent proteins that did not meet the setting criteria. (**C**) The expression of adiponectin receptor 1 (AdipoR 1) protein in hippocampus. (**D**) Quantitative analysis of the AdipoR 1 protein according to the Western blot results. (**E**) Immunofluorescence results of the expression of AdipoR 1 protein in hippocampus. The white arrows indicate the AdipoR 1 protein. Scale bar: 10 μm. AdipoR 1: adiponectin receptor 1. * *p* < 0.05.

**Figure 2:**
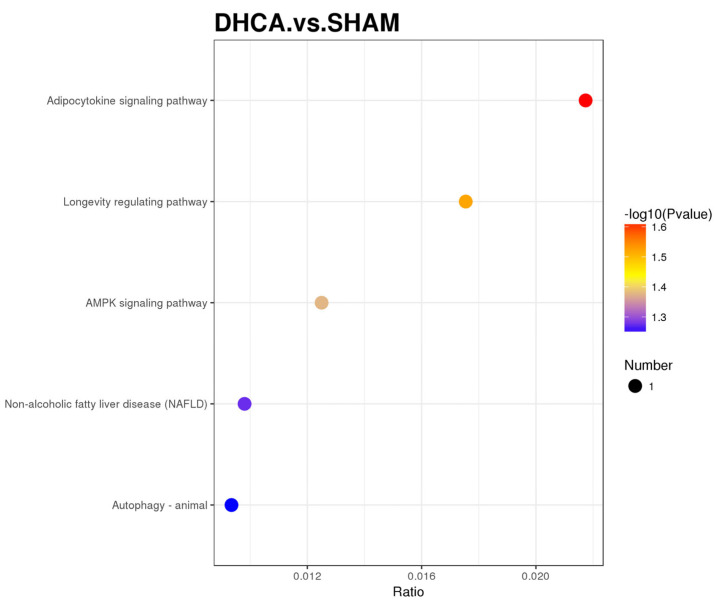
KEGG pathway enrichment analysis of upregulated proteins in the hippocampus of DHCA rats.

In order to explore the mechanism of central nervous system injury after the DHCA procedure, the different expression proteins of hippocampus which are sensitive to ischemia and hypoxia were investigated. After DHCA, 19 different expression proteins were found—5 proteins were upregulated and 14 proteins were downregulated (Figure 1A,B). Adiponectin receptor 1 (AdipoR 1) protein was among the five upregulated proteins. The KEGG annotation showed that the adipocytokine signaling pathway and the AMPK signaling pathway were upregulated (Figure 2). Consistently, the AdipoR 1 is involved in the adipocytokine signaling pathway, which contains the activating of AMPK protein [12]. The results of proteomic analysis were confirmed by Western blot (Figure 1C,D) and immunofluorescence (Figure 1E), respectively, from the perspective of quantification and localization.

### 3.2. Effects of AdipoRon on Physiological Parameters during CPB with DHCA

**Figure 3:**
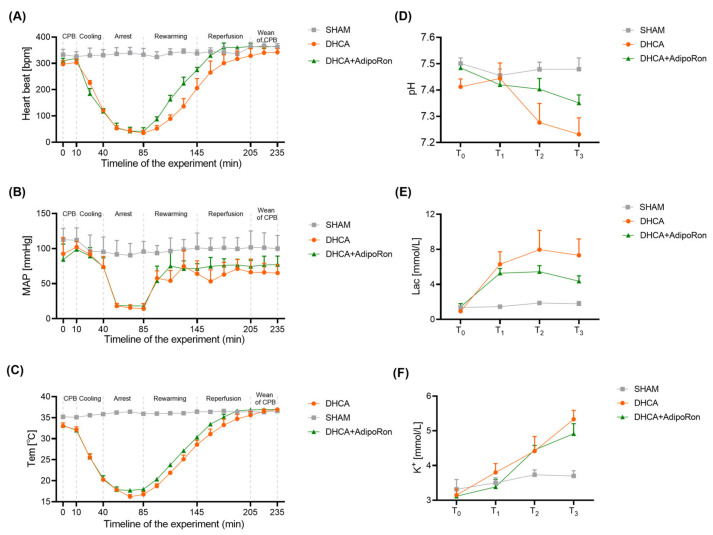
Impact of AdipoRon on cardiocirculatory recovery and blood gas results after DHCA in rats. Heart rate (**A**), mean arterial blood pressure (**B**), and temperature (**C**) were monitored throughout the entire procedure. Blood gas analysis was performed at predefined time-points for both groups. pH (**D**), plasma concentration of lactic acid (**E**) and potassium (**F**) were measured and compared. Rats in the sham group were only anesthetized, cannulated and heparinized. The time points shown in the sham group are relative and no DHCA procedures have been performed. Vital signs and the results of the blood gas analysis are shown as real values (n = 6). Data are presented as the mean ± standard error of median (SEM). Student’s *t* test was used to compare the differences between the DHCA group and the AdipoRon-treated DHCA group at each time point. MAP: mean arterial blood pressure; Tem: temperature; Lac: lactic acid; DHCA: deep hypothermia circulatory arrest group; SHAM: sham group. T_0_: 10 min after the initiation of CPB, T_1_: 45 min after the DHCA, T_2_: 60 min after rewarming, and T_3_: 60 min after the reperfusion. *p >* 0.05 was not shown.

To observe the therapeutic effects of AdipoRon in vivo, we constructed DHCA rat models and administrated the DHCA rats with AdipoRon. The initial 10 min after the CPB establishment is an adapting stage for rats to the CPB circuit. In this process, the HR, MAP, body temperature, blood pH, concentration of lactic acid and potassium were similar among the sham, DHCA, and AdipoRon-treated DHCA rats (Figure 3). Then, the body temperature decreased to 20 °C with a 30 min cooling procedure in which the HR decreased from 300 to 100 beats per minute (bpm) in both DHCA and AdipoRon-treated DHCA rats. When the body temperature reached 20 °C, the rat’s blood was drained into the reservoir, and the heartbeat arrested along with a sharp drop of MAP (Figure 3A,B). It is worth noting that AdipoRon administration attenuated the increased lactic acid (Figure 3E) in DHCA rats during the cardiac arrest.

After the 45 min DHCA, the blood in the reservoir was slowly (1 mL/min) re-administered to the rat body and the rewarming period began. To prevent the sharp increase in body temperature, another 60 min reperfusion was employed for a gradual increase to normal body temperature (Figure 3C). During the rewarming period, we found that AdipoRon administration improved the recovery speed of temperature and HR in DHCA rats. Additionally, in the whole period of rewarming and reperfusion, HR, MAP, and body temperature in AdipoRon-treated DHCA rats were higher than in DHCA rats. Furthermore, AdipoRon significantly rescued the decreased blood pH and increased lactic acid in DHCA rats (Figure 3D,E). It is worth noting that AdipoRon attenuated the increased K+ in DHCA rats after the cardiac arrest (Figure 3F). Although the indicators at each time point are not statistically different, the trends show AdipoRon could increase the adaptability of cardiovascular system to the DHCA procedure and alleviate the accumulation of lactic acid caused by DHCA.

### 3.3. AdipoRon Has an Anti-Inflammatory Effect in Rats

**Figure 4:**
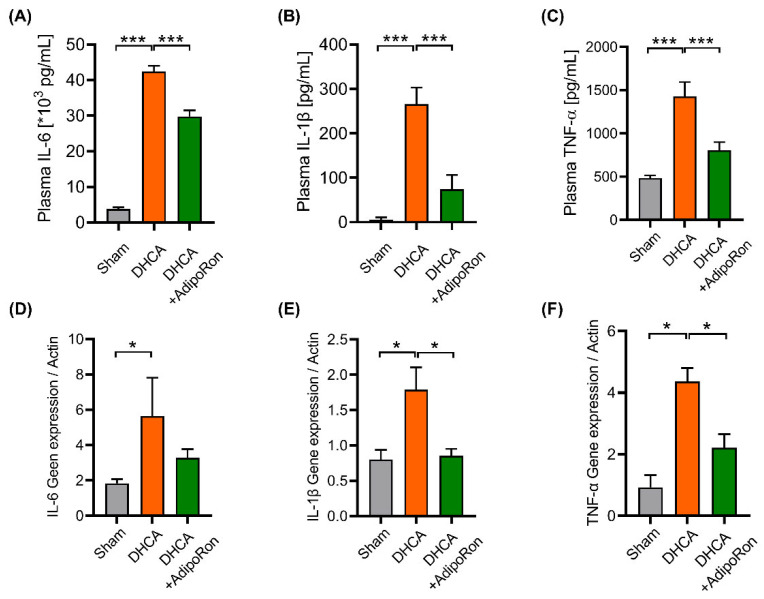
Assessment of anti-inflammation effects of AdipoRon in DHCA rats. Blood samples were collected into the tubes containing EDTA at the end of the surgical procedure and then centrifuged (3000 rpm for 20 min at 4 °C) within 30 min. The upper layers of plasma were stored at −80 °C for analysis. Concentrations of IL-6 (**A**), IL-1β (**B**) and TNF-α (**C**) were calculated based on a comparison of standard concentrations provided by the ELISA kit (n = 6). Rats were sacrificed and the brain was collected at the end of the surgery. mRNA expression of the IL-6 (**D**), IL-1β (**E**) and TNF-α (**F**) in the brain was measured relative to Actin by quantitative real-time polymerase chain reaction. Data are presented as the mean ± error of median (SEM). Student’s *t* test was used to compare the differences between two groups. IL-6: interleukin 6, IL-1β: interleukin 1β; TNF-α: tumor necrosis factor α; DHCA: deep hypothermia circulatory arrest group; SHAM: sham group. * *p* < 0.05 and *** *p* < 0.001.

Acute inflammation is considered a critical component in the pathological process after DHCA [8,23]. Proinflammatory cytokines may significantly influence the development and maintenance of inflammation response. Therefore, we tested the concentrations of three major proinflammatory cytokines (IL-1β, IL-6, and TNF-α) in plasma and the cerebral cortex at the end of the DHCA procedure to investigate the anti-inflammatory effect of AdipoRon in vivo. After surgery, compared with sham rats, plasma concentrations of IL-6, IL-1β, and TNF-α in DHCA rats were significantly increased (Figure 4A–C), verifying the systemic inflammatory responses following DHCA. Meanwhile, the concentrations of IL-6, IL-1β, and TNF-α in AdipoRon-treated DHCA rats significantly decreased (*p* < 0.001). Due to the low concentration of cytokines in the cortex, we analyzed the transcription of cytokine genes and found that the mRNA of IL-1β (Figure 4E) and TNF-α (Figure 4F) in the AdipoRon group were significantly lower than that in the DHCA group, which means AdipoRon attenuates the acute neuroinflammation induced by DHCA.

### 3.4. AdipoRon Inhibited Neuroinflammation Following DHCA Involving AMPK Phosphorylation and NF-κB Downregulation

**Figure 5:**
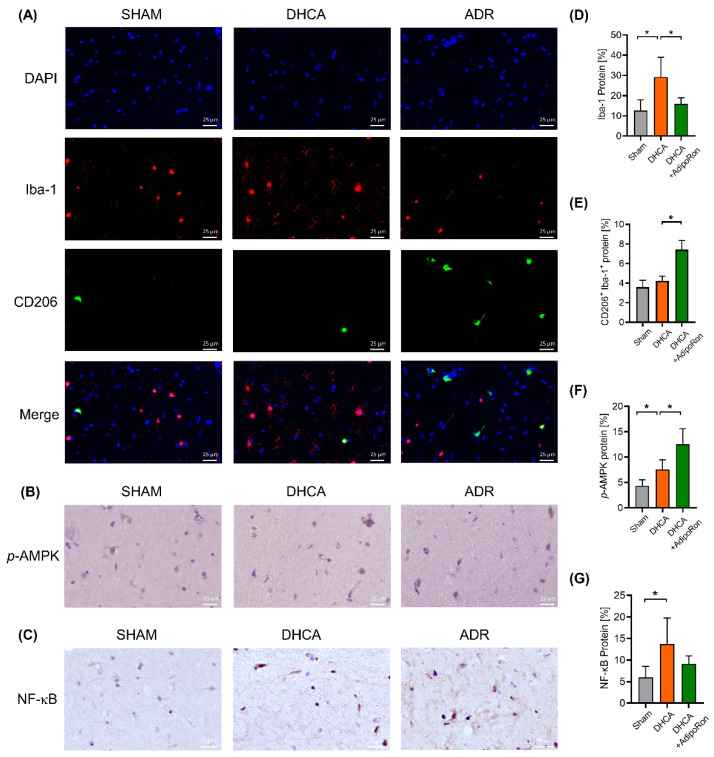
Impact of AdipoRon on Iba-1, CD206, p-AMPK, and NF-κB protein in rats’ hippocampus. Rats with or without AdipoRon treatment that underwent DHCA were sacrificed and the hippocampus tissue was collected at the end of the surgery. (**A**): Immunofluorescence detection of ionized calcium-binding adaptor molecule-1 (Iba-1) and CD206 in hippocamus tissue of the sham group, the DHCA group and the AdipoRon-treated DHCA group. (**B**,**C**): Immunohistochemistry detection of phosphorylation status of AMP-activated protein kinase (p-AMPK) and nuclear factor κB (NF-κB) protein in hippocampus tissue of the sham group, the DHCA group and the AdipoRon-treated DHCA group. A total of 6 animals per group were examined. Representative images are depicted. Scale bar: 25 μm. (**D**–**G**): Quantification of Iba-1, CD206, p-AMPK, NF-κB expressed as the mean expression intensity. The *y*-axis shows the percentage of positive cell area to total cell area. Student’s *t* test was used to compare the differences between the DHCA group and the AdipoRon-treated DHCA group. Results are presented as the mean ± SEM, * *p* < 0.05.

**Figure 6:**
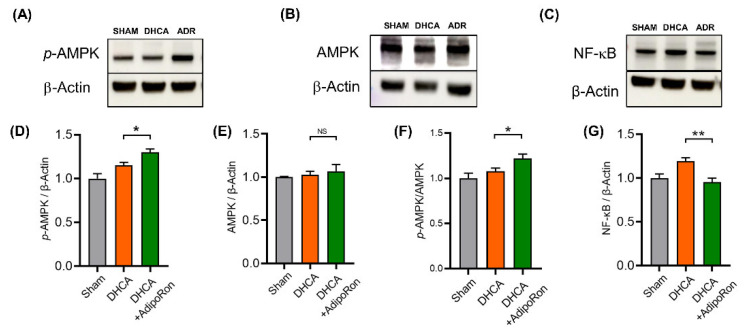
AdipoRon modulates activation of AMPK and downregulates NF-κB protein in rat hippocampus. Expression of (**A**) phosphorylation status of AMP-activated protein kinase (p-AMPK; molecular weight: 62 kDa), (**B**) AMPK-activated protein kinase (AMPK; molecular weight: 62 kDa), (**C**) nuclear factor κB (NF-κB; molecular weight: 65 kDa) in rat hippocampus of AdipoRon- and vehicle treated rats was measured by immunoblot. An amount of 30 μg protein of each sample was used for gel electrophoresis. Column bars indicated quantified levels of target protein/Actin expression ratios (**D**–**G**), and the results are relative values whereby the expression level of the sham control was normalized to 1.0. n = 6 in each group. Results are presented as the mean ± SEM. Student’s *t* test was used to compare the differences between the DHCA group and the AdipoRon-treated DHCA group. * *p* < 0.05 and ** *p* < 0.01*. NS: p >* 0.05.

The activation of microglia is considered a rapier in neuroinflammation according to its polarizing states [25]. The M1 microglia release proinflammatory cytokines, while M2 microglia play anti-inflammatory neuroprotective roles by promoting neural repair and regeneration. Therefore, we here used the microglial activating biomarker Iba-1 and the M2 microglia biomarker CD206 to observe the microglial states after DHCA and AdipoRon administration. Compared with sham rats, the expression of Iba-1 protein was increased in DHCA rats, indicating the activation of hippocampal microglia (Figure 5A). In AdipoRon-treated DHCA rats, hippocampal microglia were inhibited compared with the DHCA rats (Figure 5A,D). The expression of CD206 protein in Iba-1^+^ microglia was similar in sham rats and DCHA rats, while it was significantly increased in AdipoRon-treated DHCA rats (Figure 5A,E). Considering the decreased pro-inflammatory cytokines in AdipoRon-treated DHCA rats (Figure 4), we speculated that AdipoRon triggered anti-inflammatory polarization of microglia. Thus, the M2-inducer p-AMPK and M2-inhibitor NF-κB were then tested with IHC and Western blot. As predicted, IHC results showed that AdipoRon treatment indued an extensive increase in p-AMPK and decrease in NF-κB in the hippocampus of DHCA rats (Figure 5B,C). These results were also confirmed by Western blot (Figure 6). Consequently, AdipoRon inhibited neuroinflammation following DHCA involving AMPK phosphorylation and NF-κB downregulation.

### 3.5. AdipoRon Attenuates Neuroinflammation in BV2 Cells

**Figure 7:**
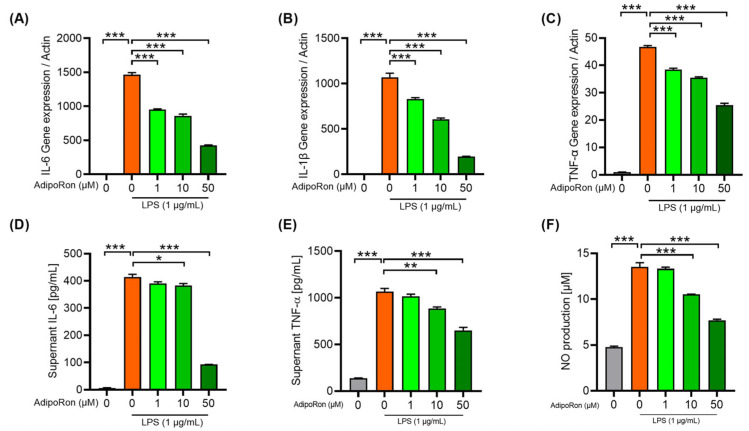
Anti-neuroinflammation effect of AdipoRon on BV2 cells. mRNA expression of the IL-6 (**A**), IL-1β (**B**) and TNF-α (**C**) were measured relative to Actin by quantitative real-time polymerase chain reaction. BV2 cells were preincubated with AdipoRon (0–50 μM) or vehicle (dimethyl sulfoxide) for 1 h before stimulation with lipopolysaccharide (1 μg/mL) for 4 h. Concentration of IL-6 (**D**) and TNF-α (**E**) in culture supernatants was quantified by ELISA (n = 3). BV2 cells were preincubated with AdipoRon (0–50 μM) or vehicle for 1 h before stimulation with lipopolysaccharide (1 μg/mL) for 6 h. Concentration of NO (**F**) in culture supernatants was quantified by a Nitric Oxide Assay Kit (n = 3). BV2 cells were preincubated with AdipoRon (0–50 μM) or vehicle for 1 h before stimulation with lipopolysaccharide (1 μg/mL) for 24 h. Results are presented as the mean ± SEM. Differences between experimental groups were analyzed statistically by performing one-way ANOVA followed by Bonferroni’s multiple comparison tests. IL-6: interleukin 6, IL-1β: interleukin 1β; TNF-α: tumor necrosis factor α. * *p* < 0.05, ** *p* < 0.01, and *** *p* < 0.001.

Microglia are the foremost and earliest inflammatory cells participating in the pathological process of neuroinflammation. Here, we used the BV2 cell, a commonly used cell line for the LPS-induced microglial inflammation model [26], to investigate the impact of AdipoRon on neuroinflammation in vitro. Due to the inconsistent reports, we firstly explored and confirmed that 1 μg/mL LPS could significantly induce the inflammatory response in BV2 cells. Additionally, to clarify the anti-inflammatory effects of AdipoRon, we assessed mRNA and protein levels of the downstream proinflammatory cytokines. Results showed that 0–50 μM AdipoRon dose-dependently inhibited the expression (Figure 7A–C) and secretion (Figure 7D,E) of proinflammatory cytokines (IL-6, IL-1β, TNF-α). Further, we found that 0–50 μM AdipoRon dose-dependently inhibited the NO production in the supernatant of LPS-induced BV2 cells (Figure 7F), which indirectly reflected a decreased expression of inducible nitric oxide synthase (iNOS). Taken together, we confirmed the anti-neuroinflammatory effect of AdipoRon in vitro.

### 3.6. AdipoRon Inhibits Neuroinflammation in BV2 Cells via Activating AMPK Protein and Downregulating NF-κB Protein

**Figure 8:**
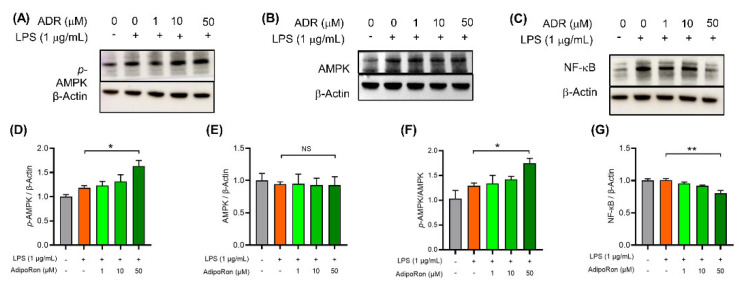
The expression of AMPK, p-AMPK and NF-κB protein of BV2 cells. Expression of (**A**) phosphorylation status of AMP-activated protein kinase (*p*-AMPK; molecular weight: 62 kDa), (**B**) AMPK-activated protein kinase (AMPK; molecular weight: 62 kDa), and (**C**) nuclear factor κB (NF-κB; molecular weight: 65 kDa) in BV2 cells was measured by immunoblot. BV2 cells were preincubated with AdipoRon (0–50 μM) or vehicle (dimethyl sulfoxide) for 1 h before stimulation with lipopolysaccharide (1 μg/mL) for 1 h. An amount of 10 μg protein of each sample was used for gel electrophoresis. Column bars indicated quantified levels of target protein/Actin expression ratios (**D**–**G**), and the results are relative values whereby the expression level of the sham control was normalized to 1.0. n = 3 in each group. Results are presented as the mean ± SEM. LPS: lipopolysaccharide; ADR: adipoRon. * *p* < 0.05 and ** *p* < 0.01*. NS: p >* 0.05.

AMPK is a heterotrimeric serine/threonine-protein kinase and its phosphorylation involves the anti-inflammatory M2 polarization of microglia [27,28]. To investigate whether AMPK activation participated in the anti-inflammatory process of AdipoRon, we then tested the p-AMPK expression levels in LPS-induced BV2 cells. AMPK activation or phosphorylation is a rapid and short-term process. Therefore, we tried to observe the expression of p-AMPK protein at 1, 2, and 24 h after AdipoRon administration. At 1 h after the intervention, 0–50 μM AdipoRon dose-dependently upregulated the expression of p-AMPK in LPS-stimulated BV2 cells (Figure 8A,D,F), which is consistent with the decreased proinflammatory cytokines. Interestingly, we did not observe any difference in p-AMPK protein expression between different groups after 2 h of AdipoRon administration (Supplementary Figure S2A). Additionally, after 24 h, the expression level of p-AMPK protein was decreased in AdipoRon-treated BV2 cells (Supplementary Figure S2B). Further, AdipoRon did not influence the expression of AMPK protein (*p* > 0.05, Figure 8B,E).

Different from p-AMPK, the NF-κB is a transcriptional factor and the activation of NF-κB promotes the proinflammatory M1 polarization of microglia [29,30]. Naturally, NF-κB in BV2 cells was significantly increased after LPS stimulation. To further evaluate the anti-inflammatory ability of AdipoRon, we also assessed the expression of NF-κB and found a dose-dependent decrease in NF-κB in AdipoRon-treated LPS-stimulated BV2 cells (*p* < 0.01, Figure 8C,G). These results collectively demonstrated that AMPK and NF-κB participated in the anti-inflammatory process of AdipoRon.

## 4. Discussion

DHCA can cause neuroinflammation and increase the incidence of post-operative neurological complications. In the present study, we firstly found that the AdipoR 1 protein was upregulated in hippocampus after the DHCA procedure via the proteomic analysis. Considering AdipoRon is an adiponectin receptor agonist, we used it in the DHCA rat model to investigate its therapeutic effect and the results showed that AdipoRon could attenuate the acute neuroinflammation induced by DHCA in vivo *and* in vitro and inhibit the activation of microglial cells in the hippocampus, in which it triggered anti-inflammatory polarization involving AMPK phosphorylation and NF-κB downregulation. This study is the first to clarify the inhibitory function of AdipoRon on DHCA-induced neuroinflammation, which is helpful to enrich understanding of AdipoRon and the adipocytokine signaling pathway, and contributes to the development of compounds with similar structures.

Using the DHCA rat model, we found that DHCA can induce SIRS, increase the concentration of proinflammatory cytokines in plasma, and promote the activation of microglia in the hippocampus, accompanied by the increase in *p*-AMPK and NF-κB. The opposite roles of NF-κB and *p*-AMPK in microglial polarization indicate the onset of both pro-inflammatory and compensatory anti-inflammatory processes after DHCA. After the administration of AdipoRon, hippocampal *p*-AMPK further increased, and NF-κB decreased, suggesting an anti-inflammatory M2 polarization of microglia. This is consistent with the previously reported regulatory role of AdipoRon on AMPK and NF-κB [11] and also confirmed by the increased CD206^+^ IbA1^+^ cells in the hippocampus. Further, we considered that both direct and indirect inhibition might account for the downregulation of NF-κB following AdipoRon administration because NF-κB is also a downstream transcriptional factor of *p*-AMPK [31].

We speculated that the above two states existed in the activation of microglia during DHCA. The inflammatory response induced by the DHCA procedure results in activation of the pro-inflammatory state of microglia and a compensatory inhibitory state of inflammation. Recent studies have shown that AdipoRon has the ability to attenuate inflammation via promoting the phosphorylation of AMPK [32]. Our results demonstrated that AdipoRon could inhibit microglial activation by promoting AMPK phosphorylation and inhibiting NF-κB protein expression. AdipoRon inhibits the activation of microglia (M1 state) and promotes their transition to an anti-inflammatory state (M2 state). NF-κB protein is downstream of the AMPK in the AMPK signaling pathway. Meanwhile, it has been demonstrated that adiponectin/AdipoRon could inhibit the NF-κB pathway independently [33]. The downregulation of NF-κB protein is the result of both AdipoRon’s action on AMPK protein and AdipoRon’s exclusive participation in the NF-κB signaling pathway.

The transcription factor NF-κB exists as homo- or heterodimers bound to IκBα. IκBα kinase engages in phosphorylation of NF-κB and ubiquitin-mediated degradation of this product via the proteasome pathway [34,35]. NF-κB downregulation was associated with its cytoplasmatic inhibitor IκBα upregulation [36]. Nuclear factor erythroid 2-related factor 2 (Nrf2) is a transcription factor that regulates cellular defense and is involved in many cellular processes, including inflammation [37]. NF-κB has an impact on the activation of the Nrf2 transcription [38]. The DHCA procedure can decrease the Nrf2 and its downstream enzymes NADPH quinine oxidoreductase-1 (NQO-1) and haemoxygenase-1 (HO-1) [39]. At the same time, AdipoRon induced a significant elevation in epididymal adipose tissue content of Nrf2 while evaluating its anti-diabetic effects [40]. Taken together, the changes in phosphorylated NF-κB, IκBα, Nrf2, NQO-1 and HO-1 proteins under AipoRon are still worth of further study.

Based on our results, the frequency and dosage of AdipoRon should be further studied in the future. In order to avoid the insignificant therapeutic effect of single intraoperative administration, AdipoRon was administered twice during the DHCA procedure [13]. In vivo, two intraoperative dosing can inhibit inflammation induced by DHCA at the end of surgery, but whether it is necessary to continue the dosing after surgery to make it play a longer effect needs further investigation. In vitro, AdipoRon significantly inhibited pro-inflammatory cytokines 6 h after administration, while it took 24 h to observe significant differential changes in NO production. These showed that the duration of the pharmacological effect produced by one dose of AdipoRon is at least 24 h. Further, AdipoRon is an orally active synthetic small-molecule agonist and previous studies have shown that oral administration enhanced cell response to oxidative stress [41]. Future studies will investigate the optimal frequency for different administration methods.

The optimal concentration of AdipoRon is also worth further discussion. Alexander et al. have found that AdipoRon has anti-inflammatory effects at 60–100 μM on inflammation induced by LPS (1 mg/mL) [13]. However, Sarah et al. believed AdipoRon did not exert anti-inflammatory effects based on their results, in which 10^−6^ M AdipoRon did not inhibit the cytokines induced by LPS (0.5 μg/mL) [42]. The effective concentration of AdipoRon seemed to correlate with the degree of inflammatory response and in our experiment, the effective concentration of AdipoRon was 0–50 μM under the stimulation of 1 μg/mL LPS. These differences may be due to the different cell types treated by AdipoRon in different studies.

In addition to the ischemia-reperfusion model we provided, AdipoRon exhibited anti-inflammatory effect under a number of experimental conditions. It has been proved that AdipoRon can significantly inhibit post-ischemic cardiomyocyte apoptosis and improve cardiac functional recovery after reperfusion [43]. In the central nervous system, AdipoRon could not only attenuate neuroinflammation after intracerebral hemorrhage but could also improve cognitive dysfunction or impaired neural stem cell proliferation in the early stage of Alzheimer’s disease via the AdioR1-AMPK pathway [21,44]. Our findings, together with those of other research teams, suggest that AdipoRon’s anti-inflammatory effect is definitive. For neuroinflammation caused by DHCA, studies have found that resveratrol and chlorogenic acid can also effectively inhibit the inflammatory response thorough the NF-κB pathway [45,46]. Taken together, it will be among the directions in the future whether the above compounds can be applied in DHCA together with a better anti-inflammation effect.

There are some limitations throughout the research process. It would be better to test the rats’ behaviors during rehabilitation among the different groups and the results will be more convincing. Further, the concentration of AdipoRon in DHCA rats was not assessed, and the concentrations of AdipoRon in plasma and brain need further investigation.

## 5. Conclusions

Our present data supported that AdipoRon inhibited DHCA-induced neuroinflammation involving activating the hippocampal AMPK/NF-κB pathway. AdipoRon might be an available clinical medicine for preventing neuroinflammation following DHCA. The results will provide a reference for a comprehensive understanding of adiponectin receptor agonists and provide new ideas for clinical development of drugs that can alleviate DHCA-related neurological complications.

## Data Availability

The data presented in this study are available on request from the corresponding author.

## Supplementary Materials

The following supporting information can be downloaded at: https://www.mdpi.com/article/10.3390/pharmaceutics14112467/s1, Figure S1: Flow chart of DHCA model in rat; Figure S2: The impact of AdipoRon on the activation of AMPK in BV2 cell; Table S1: Specific primers for quantitative real-time polymerase chain reaction.
