# Supplementary material for: AdipoRon Inhibits Neuroinflammation Induced by Deep Hypothermic Circulatory Arrest Involving the AMPK/NF-κB Pathway in Rats
Source: Pharmaceutics. 2022 Nov 15;14(11):2467. doi: 10.3390/pharmaceutics14112467 (PMC9698032; doi:10.3390/pharmaceutics14112467)
Supplement: Supplementary file 1 [file pharmaceutics-14-02467-s001.zip › pharmaceutics-1952970-supplementary.pdf]

**AdipoRon Inhibits Neuroinflammation Induced by Deep Hypothermic Circulatory Arrest Involving the AMPK/NF-κB Pathway in Rats**

Weidong Yan[1], Sizhe Gao[1], Qiaoni Zhang[1], Jiachen Qi[1, 2], Gang Liu[1], Yuan Teng[1], Jian Wang[1], Shujie Yan[1], Bingyang Ji[1, *]

1 Department of Cardiopulmonary Bypass, Fuwai Hospital, National Center for Cardiovascular Disease, State Key Laboratory of Cardiovascular Medicine, Chinese Academy of Medical Science & Peking Union Medical College, Beijing, 100037, China.

2 Department of Pain Medicine, Beijing Tsinghua Changgung Hospital, School of Clinical Medicine, Tsinghua University, Beijing, 102218, China

## Supplemental Figures

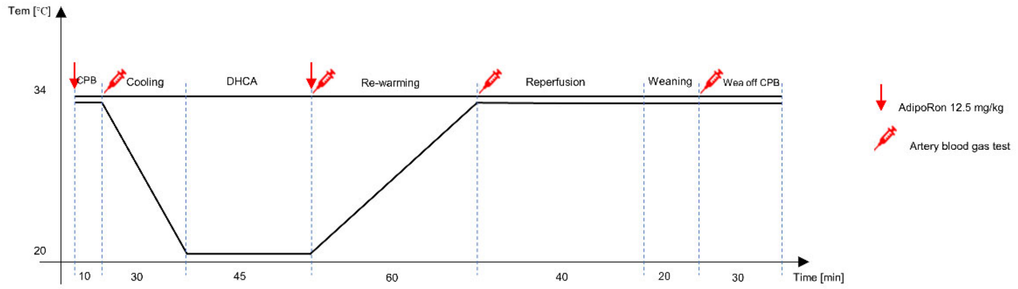


**Supplemental Figure S1: Flow chart of DHCA model in rats.**

CPB: cardiopulmonary bypass, DHCA: deep hypothermic circulatory arrest.

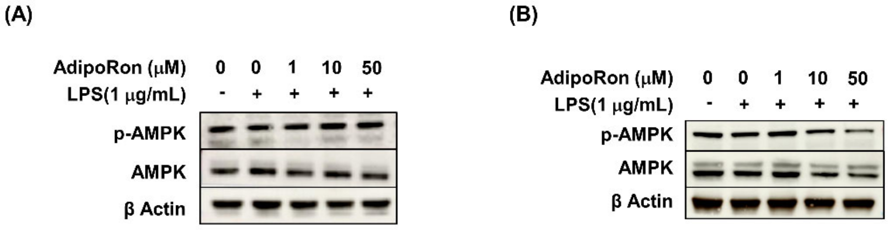


**Supplemental Figure S2: The impact of AdipoRon on the activation of AMPK in BV2 cells.**

Expression of phosphorylation status of AMP-activated protein kinase (p-AMPK; molecular weight: 62 kDa), AMPK-activated protein kinase (AMPK; molecular weight: 62 kDa) in BV2 cell were measured by immunoblot. **(A)** BV2 cell were preincubated with AdipoRon (0 – 50 μM) or vehicle (dimethyl sulfoxide) for 1 hour before stimulation with lipopolysaccharide (1 μg/mL) for 2 hours. **(B)** BV2 cell were preincubated with AdipoRon (0 – 50 μM) or vehicle (dimethyl sulfoxide) for 1 hour before stimulation with

lipopolysaccharide (1 μg/mL) for 24 hours. 20 μg protein of each sample was used for gel electrophoresis.

**Supplemental Table**

**Supplemental Table S1. Specific primers for quantitative real-time polymerase chain reaction.**

| Inflammatory factor gene | Sequence |
|---|---|
| Mouse IL-6 | Forward: TAGTCCTTCCTACCCCAATTTCC |
| | Reverse: TTGGTCCTTAGCCACTCCTTC |
| Mouse IL-1β | Forward: GCAACTGTTCCTGAACTCAACT |
| | Reverse: ATCTTTTGGGGTCCGTCAACT |
| Mouse TNF-α | Forward: CTGAACTTCGGGGTGATCGG |
| | Reverse: GGCTTGTCACTCGAATTTTGAGA |
| Mouse Actin | Forward: GGACTCCTATGTGGGTGACG |
| | Reverse: CTTCTCCATGTCGTCCCAGT |
| Rat IL-6 | Forward: ACAAGTCCGGAGAGGAGACT |
| | Reverse: GAATTGCCATTGCACAACTCT |
| Rat IL-1β | Forward: CAGCTTTCGACAGTGAGGAGA |
| | Reverse: TGTCGAGATGCTGCTGTGAG |
| Rat TNF-α | Forward: GATCGGTCCCAACAAGGAGG |
| | Reverse: CTTGGTGGTTTGCTACGACG |
| Rat Actin | Forward: CCCGCGAGTACAACCTTCTTG |
| | Reverse: TCATCCATGGCGAACTGGTGG |
